# Perceptions of dental health professionals (DHPs) on job satisfaction in Fiji: a qualitative study

**DOI:** 10.1186/s12913-022-08620-z

**Published:** 2022-10-18

**Authors:** Samantha Kumar, Masoud Mohammadnezhad

**Affiliations:** 1 School of Dentistry and Oral Health, Fiji National University, Suva, Fiji; 2 School of Nursing and Healthcare Leadership, University of Bradford, Bradford, UK

**Keywords:** Job satisfaction, Determinants, Dental Health Professionals, Perceptions, Fiji

## Abstract

**Background:**

Reviewing job satisfaction is crucial as it has an impact on a person’s physical and mental wellbeing, as well as leading to a better organizational commitment of employees that enhances the organizations succession and progress as well as better staff retention. This study aimed to explore the perceptions of job satisfaction amongst Dental Health Professionals (DHPs) in Fiji and associated factors.

**Methods:**

This study used a phenomenological qualitative method approach commencing from August to November, 2021. The target group for this study were the DHPs who provide prosthetic services. This study was conducted among DHPs from 4 purposively selected clinics in Fiji. A semi- structured open-ended questionnaire was used to collect data. Thematic analysis was used to transcribe and analyze the audio qualitative data collected from the interviews.

**Results:**

Twenty-nine DHPs took part in the in-depth interview and the responses were grouped into three themes. The findings from the study indicate that DHPs are most satisfied with their teamwork and the relationship they have with their colleagues and co-workers, followed by the nature of the work and the supervision they received. The participants indicated that they were less satisfied with professional development opportunities and least satisfied with their pay and organizational support they receive.

**Conclusion:**

The results of this study have identified gaps and areas for improvement of job satisfaction for DHPs who provide prosthetic services in Fiji such as need for more career and professional development pathways, improved infrastructure to support prosthetic service delivery in Fiji and improve remuneration for DHPs. Understanding the factors that affect satisfaction levels and being able to act accordingly are likely to lead to positive outcomes both for DHPs and their organization.

**Supplementary Information:**

The online version contains supplementary material available at 10.1186/s12913-022-08620-z.

## Background

In any institution, the most valuable asset is human resources making job satisfaction as one of the most important factors for efficiency and productivity of a sustainable service delivery [1]. Job satisfaction can be defined as having a “positive emotional state resulting from the appraisal of one’s job or job experience and is related to many aspects of patient care and outcomes of health system as well as to contentment in general life and job related performance” [2]. Reviewing job satisfaction is crucial as it has an impact on a person’s physical and mental wellbeing. It also leads to improved organizational commitment of employees that enhances the organizations success and progress as well as increased staff retention. Job satisfaction is the essence of any successful dental practice [3].

Dentistry can be a very stressful profession thus to encourage dental professionals to provide high quality service, assessing job satisfaction becomes important for understanding how work environmental factors impact it [4]. According to Gilmour et al. [5] dentistry has frequently been described as a stressful occupation, and is associated with greater incidence of ill health, alcoholism, and suicide than other medical professions. Different levels of job satisfaction have been observed in different counties such a more than 80% of Australian and Lithuanians dental professional reported being highly satisfied, while the Koreans and Egyptians had and an overall average job satisfaction of 51% and the Iranians being the least satisfied having a mean score of 1.5 out of 5 indicating they may have a very stressful work environment [6–10]. Nikolovska et al. [11] found that dental professionals in Macedonia showed overall low levels of job satisfaction among the public and private dental practitioners due to high stress levels and that private practitioners were the most satisfied compared to public practitioners. A similar result was seen in study in the United Arab Emirates (UAE) where private sector dental professionals had a higher level of satisfaction compared with the public sector [12]. Job satisfaction may be generalized as a person’s attitude towards their profession. People who experience high levels of satisfaction are likely to have a more positive outlook towards their profession in comparison to those who experience low levels of satisfaction [13].

Tuisuva et al. [14] characterized dentistry in Fiji and the region as ‘Pacific setup” which is a community-based approach where daily functions are limited by malfunctioning equipment’s, lack of quality resources and inadequate remuneration for staff, poor working environments which are indicators for low job satisfaction level. Although there is extensive literature on job satisfaction, there is little to none research on job satisfaction amongst dental professionals in Fiji, in particular dentists and dental technicians. Therefore, this study aimed to explore the perceptions and factors affecting job satisfaction among Dental Health Professionals (DHPs) in Fiji. In any institution, the most valuable asset is human resources making job satisfaction as one of the most important factors for efficiency and productivity of a sustainable service delivery. To promote the quality of life among DHPs it is important to consider factors affecting their job satisfaction where they are working and providing service to patients. This study is trying to address the Sustainable Development Goal (SDG) 3 to “ensure healthy lives and promote well-being for all at all ages”. The findings of this study will be helpful to policymakers to design plans in order to increase the level of job satisfaction of DHPs by highlighting areas where there is a need for improvement in services. This will also be helpful in identifying the need for program development for dental academic institutes.

### Conceptual framework

**Fig. 1:**
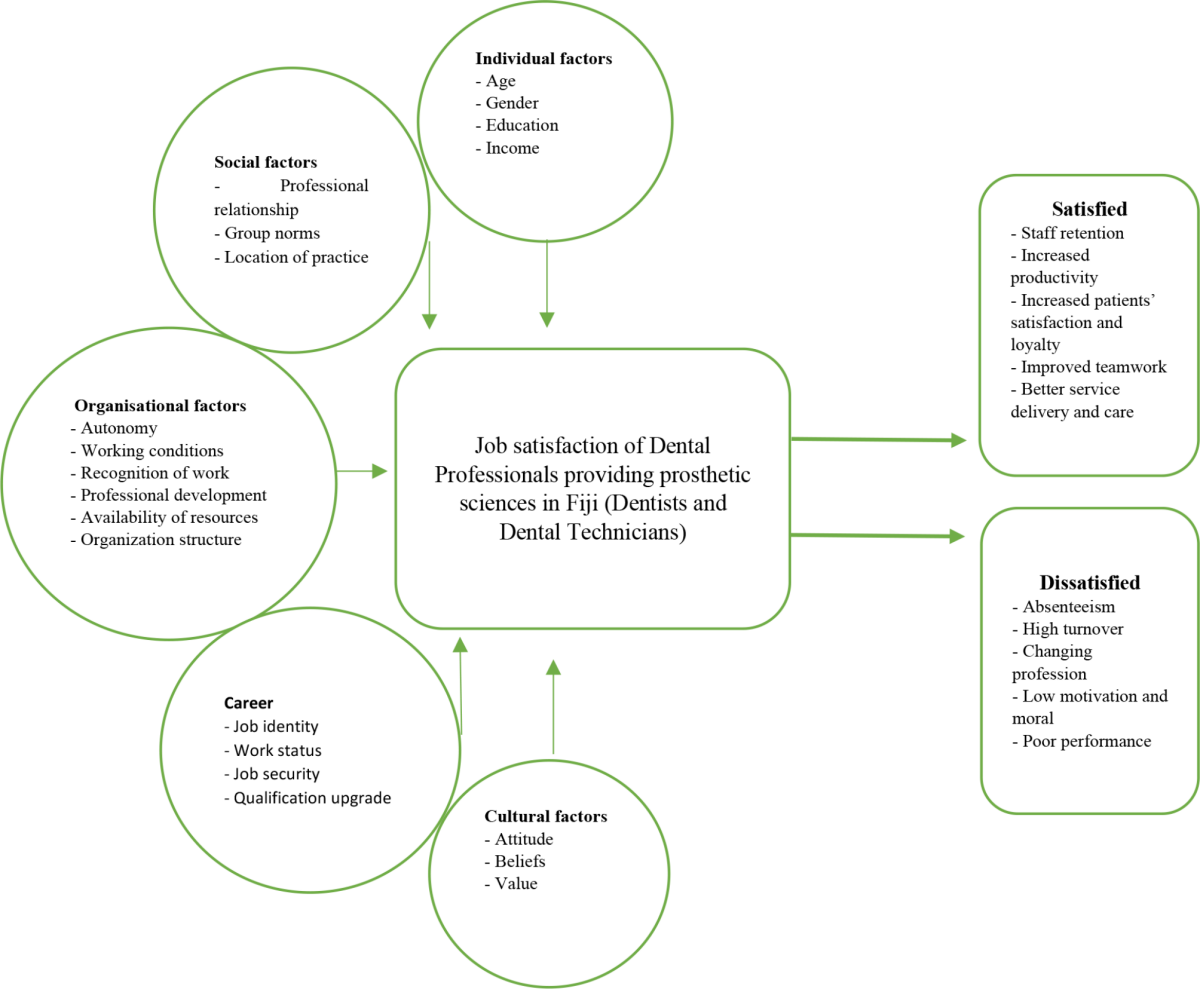
Conceptual framework

A coherent conceptual framework is constructed to elaborate the theoretical relationship between job satisfaction factors and employee commitment to organizational culture as shown in Fig. 1. This conceptual framework summarizes the variables that affect job satisfaction among dental professionals. This relationship will be tested and verified based on this research.

Factors categorized as personal and organizational determinants are associated with job satisfaction and can influence a person’s level of satisfaction and organizational commitment [1].

Personal determinants include age, gender, and level of education. Gender based differences is a determinant of job satisfaction, female practitioners ascribe more importance to social factors, while males place greater value on income, progression and other extrinsic aspects [15]. Older practitioners are generally happier with their jobs than younger employees, while people who are more experienced in their jobs are more highly satisfied than those who are less experienced.

Organizational factors include nature of work, income, interactions and career development which includes autonomy, working conditions, recognition of work, professional development, availability of resources, and organization structure. Autonomy given to employees allow them to feel confident and competent thus allowing then to make a positive impact on the organization. If employees feel that the work done by them is meaningful, has significance and is recognized, they will be satisfied. Any person would want a pay system that they perceive as just, unequivocal, and in line with their expectations. Satisfaction is likely to result when one’s income is seen as fair based on job demands, individual skill level, and in accordance with community pay standards. In any organization, employees seek fair promotion policies and practices that provide opportunities for personal growth, additional responsibilities, and improved social status. Individuals who recognize that promotional decisions are made in a fair and just manner, experience satisfaction with their jobs. Work environments that support personal comfort, growth and good job performance are highly desirable. Most employees also prefer working closer to home, in a clean and modern facility with adequate staff and resources may result in satisfaction or dissatisfaction if work environment is not suitable. Practices with more-satisfied employees tend to be more productive than those with less-satisfied employees [16].

Professional relationships, location of practice and quality of interaction indicating a need for healthy work interaction and professional networking which is a major cause of satisfaction. Studies have found that employees feel satisfaction when superior staff understands them, is friendly and approachable, compliments good performance, listens to employees’ opinions, and shows a personal interest in them. On the contrary, practices with toxic work culture where staff feel undermined, disrespected, undervalued and threated, are generally dissatisfied [17].

Finally, professional factors include job identity, work status, job security and qualification. Employees with renewed contracts/ permanent placement have a sense of job security, feel more satisfied. This gives them job identity and status. Career development and qualification upgrade incentives provide opportunities for staff enhance their skills and knowledge which in turn benefits both the organization and staff and are satisfying aspects of workplaces [18].

Job satisfaction can be an important indicator of how DHPs feel about their jobs add value to their practice such as retentions of skilled staff, increase productivity, reduce turnover, enhanced patient satisfaction and loyalty, improve teamwork, high quality service delivery and care. On the other hand, dissatisfaction can cause employees to have frequent absenteeism, high turnover, thoughts of changing profession, low motivation and moral, high stress levels and poor performance. All these factors are interrelated and has an implication on the other which determines satisfaction or dissatisfaction.

## Methods

### Study design and setting

This study applied a descriptive qualitative study design among DHPs of Fiji which allowed for much richer and detailed responses from subjects being studied [19]. This study was conducted among the DHPs based at the dental clinics of the Ministry of Health and Medical Services (MoHMS) that provide prosthetic services located in Fiji namely Nakasi Dental Clinic, Lautoka Hospital, Labasa Hospital, and at the FNU Dental Clinic in Suva. These clinics had been purposively selected as they had an attached established laboratory support with registered staff under the Fiji Dental Council (FDC). In this study, the Consolidated Criteria for Reporting Qualitative Studies (COREQ) guidelines were followed to ensure rigor (Appendix 1).

### Sampling

A non-probability sampling design [20] in the form of a homogeneous purposive sampling method was used to gather data as target population information. This study included all the DHPs in Fiji. Those DHPs who met the study criteria were included in this study as study sample. They were Dentists and dental technicians registered under the FDC, Minimum work experience of 6 months, working at the selected clinics in the time of data collection, must be attached to the dental prosthetic clinic/ lab, and DHPs of both genders or any ethnicity. Those who worked in private dental clinics or were not willing to participate in this study were excluded.

A total of 29 DHPs were approached for the in-depth interviews. The sample participants included 18 dental technicians and 11 dentists. The final sample size was based on the data saturation.

### Data collection tool

The data had been collected through a semi-structured open-ended questionnaire in the form of an interview via zoom with participants. The questions were developed based on the literatures and study research questions which comprised of two sections including (Appendix 2):

1) Demographic variables containing five questions including: age, race, gender, qualification, years of work experience and dental specialty.

2) Seven open ended questions related to job satisfaction in the English language as all participants were literate in English which eliminated the need for translation in other languages.

### Data collection

Due to the global pandemic crisis of COVID 19, the study procedure had to be amended to accommodate the movement restrictions put in place by the Fijian government. Thus, the use of a virtual platform had been employed to aid in data collection. Ethical approval was obtained from the College Health Research Ethics Committee (CHREC) with the ID#032.21 before commencement of the study. A list of suitable participants was made by contacting the dental clinics and those that met the inclusion/ exclusion criteria were contacted via phone/email requesting their participation and date and time of interview was confirmed. An information sheet together with a consent form was emailed to them. On the day of interview, a guideline was explained, and a verbal consent was recorded before commencing the interview question on Zoom. It was made clear to the participants that their identity would not be disclosed, confidentiality will be maintained and were requested to give their frank opinions. Each interview took about 20–30 min. During the interview, the conversation was recorded and note taking was done by the principal researcher. After each interview, the resulting audio was transcribed, and notes from the interviews were reviewed to identify all references and content that related to job satisfaction. Transcripts were discussed with participants for comment and/or correction.

### Trustworthiness and reflexivity

Trustworthiness is considered a more appropriate criterion for evaluating qualitative studies. In order to ensure the process is trustworthy, four criterions had been adopted. They are credibility, transferability, dependability, and confirmability [21]. There were many strategies to address credibility that include “prolonged engagement” and member checks. In-depth interviews were conducted over the period of 1 month and each interview lasted for at least 20–30 min. All in depth interviews were recorded and transcribed by the researcher on the same day. Every step of the thesis and results was discussed with the principal supervisor.

Transferability related to the ability of the findings to be transferred to other contexts or settings. Because qualitative research is specific to a particular context, it is important a “thick description” of the particular research context is provided to allow the reader to assess whether it is transferable to their situation or not. Purposive sampling technique was used in this research and in-depth interviews were carried out until data saturation was achieved. Dependability ensured that the process is described in sufficient detail to facilitate another researcher to repeat the work. This study was developed from the early stages through a systematic search of the existing literature. Same in-depth interview questions were asked to all participants of the study and review of transcriptions were done to correct errors. All data was coded and the coding was checked and rechecked thorough. Confirmability was comparable to objectivity in quantitative studies. Here, the goal was to minimize investigator bias by acknowledging researcher predispositions. Adherence to this framework by adopting strategies, such as those outlined, to address the individual criteria supports a rigorous research process. Data was checked by the principal supervisor-MM. During the interview process, note taking of the participants comments was done by principle researcher SK.

### Research team

The research team consisted of the principle female researcher who was a master student in health services management and attended a 2-hour training for conducting interviews (SK) while being supervised and guided by principal supervisor (MM).

### Data management and analysis

The content of interview was transcribed by the main researcher. The transcribed data was then checked for sufficient quality and accuracy by main researcher before major analysis is conducted. Backup files had been created considering data management system and were updated as data preparation and analysis proceeded. The collected data were analyzed using manual thematic analysis [22]. Field notes were arranged according to codes and themes which were combined into major categories and files (word documents) will be created and labeled. The principal researcher SK read and re-read all interview transcripts and identified similar phrases and words for which numbers were assigned. The coded data that had similar characteristics was grouped together. Once grouping of similar data was completed, descriptive themes and sub themes were identified to reflect the perceptions of participants. The themes and sub-themes were checked by the principal supervisor as well MM.

## Results

Twenty-nine DHPs participated in the online one on one interview via zoom. Females made up majority of the participants (76%, n = 22). Most of the participants were in the 30–40 age range (48%, n = 14). Participants were made up of two ethnicities, ITaukei (14%, n = 4) and Indo Fijians (86%, n = 25). Looking at educational levels majority of the staff just have a baseline graduate qualification (66%, n = 19), post graduate qualification (31%, n = 9) and just 1 staff who progressed to become a specialist (3%). MoHMS is the largest employer of the participants (72%, n = 21) from Nakasi dental clinic (28%, n = 8), Lautoka dental clinic (24%, n = 7) and Labasa dental clinic (21%, n = 6) followed by FNU (28%, n = 8). In terms of years of experience, 24%(n = 7) had more than 20 years of experience. DHPs who are involved in prosthetic services were dentists (38%, n = 11) and dental technicians (62%, n = 18) (Table 1).

**Table 1:** General information of participants (n = 29)

Characteristics		Frequency	Percentage
**Gender**	Male	7	24
	Female	22	76
**Ethnicity**	Indo Fijian	25	86
	ITaukei	4	14
**Age (years)**	20–30	6	21
	31–40	14	48
	41–50	5	17
	51–60	4	14
**Qualification**	Graduate qualification	19	66
	Post graduate	9	31
	Specialist	1	3
**Work experience (years)**	Less than 20yrs	22	76
	More than 20yrs	7	24
**Location of practice**	Nakasi dental clinic	8	28
	Lautoka dental clinic	7	24
	Labasa dental clinic	6	21
	FNU	8	28
**Dental Specialty**	Dentist	11	38
	Dental technician	18	62

### Themes related to job satisfaction

Three major themes had emerged from the thematic analysis which included perception on work-related factors, barriers of job satisfaction and recommendation to promote job satisfaction. Eight sub-themes identified under three main themes including nature of work, work perspectives, work environment, limitations, Covid 19, income, professional relationships, and professional development.

Under sub-themes, codes were identified as summarized in Table 2. Participants quotations were referenced as P1 to P 29, DHPs –D (dentist) or DHPs –DT (dental technician).

**Table 2:** Themes and sub-themes identified

Themes	Sub-themes	Codes
Perception on work related factors	Nature of work	Unique job, feel satisfied, highly recommend, no job opportunities
	Work perspectives	Very hectic job, very tiring, multitasking
	Work environment	Ideal work environment, Not fully utilize skills, Outdated facilities
Barriers of job satisfaction	Limitations	Frustration due to limitations, lack of support, lack of equipment’s and material
	Covid 19	Work becomes risky, not practicing full range, reduced workload, work environment becomes frustrating
	Income	Dissatisfied with salary, return on investment, are in debt
Recommendations to promote job satisfaction	Professional relationships	Good relationship, good teamwork, supportive supervisors
	Professional development	Profession is bottle necked, take up non-clinical courses, lack of post graduate courses

### Theme 1: Perception on work-related factors

Three factors were discussed under this theme including the nature of work, work perspectives and work environment.

#### Nature of work

The first few questions asked to DHPs were based on their profession and the perceptions they have of their work. These professionals share their experience of being in this profession. Here participants described their experience of being in the profession.

Some considered their profession as a unique job due to consequence of the services they provide. It makes a good memory for the patients when they receive a service that makes them happy.

*“Being a dentist, I think it is a unique job, it is a good service that we provide to people as we maintain peoples smile, make peoples smile, so it is very satisfying to see patients happy which encourages me to provide more and better services.”* (P 24, DHPs - D)

When asked if they would recommend this career to a high school student who is deciding which career path to take, some said they would recommend based on satisfaction they get from being in the profession:

*“I highly recommend this career as it is very satisfying. You feel great when you see your patient satisfied.”* (P 10, DHPs -DT)

In contrast, majority participants said they would not recommend this career based the investment they make towards the career and lack of job opportunity.

*“Well looking at the current situation, my answer would be not as there are no job opportunities at the moment. It is not worth the time and investment put into the study.”* (P 05, DHPs -DT)

#### Work perspectives

Participants were asked to describe what their job is like most of the time. Some said that despite being a very hectic job, due to services they provide that serve people they still enjoy doing it.

*“Its quiet hectic and messy, but if you have passion for this job, it is very enjoyable as we serve the older generation in the community. I really like working in the prosthetic service.”* (P 09, DHPs -DT)

Most of the participant’s mentioned multitasking at their workplace that limit them to take care of themselves.

*“I would say it’s quiet multitasking working at the school. It starts off with teaching and clinical practice as well as research and you don’t get bored with what you are doing, you keep learning new things and a lot of problem solving.”* (P 07 DHPs –D)

#### Work environment

Here participants describe the type of work environment and conditions they are working in.

Some participants mentioned that they do not have an ideal work environment that could make a better situation for them to enjoy.

*“My work environment is not ideal; space is constrained, and we are overcrowded. Equipment’s are really old, and materials are not available readily. I am not satisfied with the type of work that we do. We are not able to apply our skills that we graduated with at MoHMS. Services are very limited.”* (P 04 DHPs - DT)

Some find their work disappointing as they are not able to fully utilize and practicing all their skills.

*“We do removable prosthesis at MoHMS. I’m not satisfied with my work but its ok for the time being. At the moment we only focus on removable prosthetics only. So, we are kind of confined to one part of prosthetics whereas the broader part we are not doing so it’s a bit disappointing not being able to practice all our skills that we graduate with.”* (P 14, DHPs -DT)

### Theme 2: barriers of job satisfaction

Different limitations, challenges faced due to Covid-19 and inadequate income were the main challenges the study participants highlighted.

#### Limitations

Most participants expressed their frustrations due to the limiting situations they face at their workplace. They don’t provide all services the y expect as well as the equipment that helps them to provide better services.

*“Currently at MOHMS we are only doing complete and partial dentures. The things we have learnt from FNU like crown and bridge and orthodontics, we are unable to practice that here because it is not being offered by the ministry. Our machines are really old and in poor conditions and we have to share equipment’s among staff which is quiet frustrating. Location and size of our lab is really small and is difficult for older patients to excess.”* (P 19, DHPs -DT)

A few participants voiced that due to lack of support from organization, there is poor advancement in service delivery:

*“As a teaching institute, we should incorporate the latest technology into our teaching but the university is quiet hesitant to assist in that and stagnates the advancements in service and delivery. There needs to be back up support for the lab and equipment breakdowns.”* (P 06, DHPs -DT)

#### Covid 19

COVID 19 also posed many work challenges. Some participant mentioned that works becomes risky due high number of patients and short of staff that directly affected the quality of service as well as their satisfaction.

*“The type of work that we do is quiet risky especially now during COVID it becomes very risky and sometimes there are increased number of patients we have to see and we are short staffed and we do work beyond our JD. So, it becomes very loaded.”* (P 15, DHPs –D)

A few participants mentioned not being able to practice a full range of dental services during Covid-19 pandemic.

*“AT the moment due to COVID, we limited to the range of services we provide. we are only just doing emergency extractions and preventive dentistry.” (P23, DHPs – D)*

Some expressed that COVID has reduced their workload due to restriction of number of patients they visit daily.

*“We see so many patients in a day that we get burnout. It is hard to continuously see 15 patients a day. now thankfully to COVID, they have really restricted the number of patients we see in a day as they also have to be swabbed. So that has given us a bit of a breather.”* (P 16, DHPs – D)

#### Income

Majority of the participants expressed their dissatisfaction with the type of salary they are getting for the amount of work they do.

*“Actually, the salary does not justify our profession. We tend to work more, and if we start thinking of that, we are not satisfied as other professions like the medical staffs get paid more.”* (P 24, DHPs -D)

Perceptions based on investments made for the career. Majority participants mentioned that their returns on their investments made for their career are not worthwhile.

*“I personally feel that the pay Dental technicians get is not enough as we invest so much into our studies, and we expect to get some returns when we start working doesn’t seem to be a good investment. Secondly, dental technology is a specialized field and not everyone can do it, I feel that we should be paid more if that is to be considered.”* (P 04, DHPs -DT)

### Theme 3: recommendation to promote job satisfaction

Participants advised the needs for changing in professional relationship as well as professional development to increase the level of job satisfaction.

#### Professional relationships

Here we look at participants expressing their perceptions on interaction they have with their supervisors and managers, colleagues and coworkers, the type of teamwork they have going on and employee involvement.

Majority of the participants expressed that they have a good relationship with their coworker which helps them to make a better situation to promote their job satisfaction.

*“For me the working environment is good, it’s very friendly, my colleagues are very good.”* (P 05, DHPs -DT)

Most participants mentioned they have a very good team with great teamwork and feedback.

*“Work environment varies day to day. Someday are good and some are not so. We have a very good team and teamwork, good feedback and communication. With teamwork, come responsibilities which should be in par with communication. Each staff have their own workload and has to be mindful that the workload we have is completed in a timely manner. We talk to each other as feedbacks are very important so that each of the staffs can improve as in any organization, feedbacks are quiet important. Another thing is that without teamwork, any school or organization will not be able to function. So, the team I’m working with, they are very good*.” (P 08, DHPs -D)

Some mentioned that their supervisors are supportive and engaged.

*“I think my current supervisor is ummm quiet supportive and engaged, empowering and makes it possible for you to progress in whatever you want to do in terms of work. He is the kind of person who takes on ideas quiet easily.” (P 11, DHPs -D)*

#### Professional development

This sub-theme describes staff development as experienced by each participant. Majority of the participants feel that there is no growth, and the profession has limited progression.

*“As technicians, I feel this profession is bottle necked. To progress we need to have more courses included in the dental technology area.”* (P 01, DHPs -DT)

They also added that due to dentistry being an expensive field, they had to take up non – clinical courses.

*“I think dentistry is such a field that is quiet expensive to begin with. So, when it comes to clinical training or post grad clinical training, there is a lot of money that is required, so financially this organization might not be very supportive and this is one thing that has hindered my development. I had to take up a non-clinical masters just because of this very reason.”* (P 07, DHPs -D)

Most participants feel there are not many professional development opportunities such as postgraduate courses available which limits staff from progressing in their career.

*“At MoHMS it is quiet limited because each specialty of dentistry has not really developed except for oral surgery in which there is a master’s program available locally. Areas like endodontics and prosthodontics there are no training available and there are no specific dental officers, so due to this there is always rotations happening so no one is really able to specialize in a particular area that they like.”* (P 12, DHPs -D)

## Discussion

Majority of the participants were satisfied with the nature of their work. They reported feeling satisfaction with providing dental services and seeing their patients satisfied. Overall, dentists have a high level of professional satisfaction and the level of satisfaction is influenced by various socio demographic and psycho-behavioral factors [14]. Participants in this study described their profession as a very hands-on work, fulfilling and unique job. According to Kaipa et al. [23], job satisfaction is a pleasurable or positive emotional state as a result of one’s job experience, that one’s job fulfils or allows for fulfilment of one’s own job values. Furthermore, he describes dentistry as rewarding profession that combines art and science, personal communication skills, with high ethical standards. This profession is a social interaction between helper and recipient in their limited job setting and with personal characteristics. This was evident in the findings of this study where participants mentioned dentistry as being a rewarding profession. Rahmi et al. [24] concluded that dentistry is a caring and prestigious profession which were important motivators for job satisfaction. Some said that they would recommend this career based on the satisfaction they get from the profession. Findings from a study conducted in Romania by Murariu et al. [25] indicates that 52% of DHPs said they would choose dentistry as a career again as they found it to be a satisfying career. On the contrary, the findings of an Indian study suggest that majority of the DHPs included in the study were partly satisfied with the dental profession as a career. This is similar to the findings of the current study where due to market saturation and poor return of investments were hindering factors that led to dissatisfaction of the career.

Taking into consideration the amount of responsibility DHPs have, dentistry is one of the most stressful of the health professions [3]. DHPs tend to address a large number of challenges faced on a daily basis: healthcare sector crisis, unsatisfied patients, lack of professionalism among the staff, unsatisfactory working conditions, stress, and pressure from the environment regarding high social and economic standards related to dental professionals [4]. All of these contribute to a strong dissatisfaction of DHPs, which is reflected in their everyday practice [8]. Additionally, some least satisfying aspects were related to the lack of motivation for continuing professional education and ongoing professional training [25]. DHPs in different work settings reported differences in satisfaction level. Those working in MoHMS were mostly dissatisfied with the work they are doing in their profession. Majority of the participants felt they are limited to only a few basic procedures they can perform they found to be disappointing. Some participant said that their job required them to multitask, play various roles in their workplace and take up administrative responsibilities which they find to be burdening. This can be related to the finding of another study stating that despite having the same education ⁄training levels, dentists in the public and private system should provide similar type of care and services [6]. However, public dentists are constrained by what the public system can afford to provide and which limits their ability to practice a full range of dentistry [26].

Work environment plays a huge role in job satisfaction and its importance cannot be underestimated [27, 28]. The working environment is one of the most crucial factors which influence the level of satisfaction as well as motivation of its employees. A study conducted by Agbozo et al. [27] found that there is direct correlation between work environment and job satisfaction. His findings concluded that work environment has a significant effect on employees’ satisfaction and emphasized the need for management to improve the work environment of employees to boost productivity. Majority participants in the study were not happy in the work environment they were in. They found it was not an ideal environment due to many reasons such as over crowdedness due to space constrains, working with poor quality materials and outdated machines and equipment’s, lack of resources available to them that inhibits good quality service delivery. A study in the United States of America (USA), reinforces this finding based on staff morale having a linear and positive relationship with organizational productivity [28].

Majority of the participants expressed their frustrations due to the limitedness of their profession in terms of range of services they can offer, lack of specialization limits the profession from progressing. These were identified as some of the major dissatisfying aspects [13]. The frustration was a result of not being able to fully utilize their skills and knowledge they graduated with at their workplace due to lack of availability of resources to cater for advanced services. This had direct implications on the range of services that could be offered at their workplace [27]. Due to only having 4 dental labs in country that serves the prosthetic needs of the general population, the centralized location of practice becomes a limiting factor not only in terms of service delivery, but it limits the opportunities for more job establishments. This leads to a higher volume of patients being tended to which in turn causes overcrowding, overworked and burnout staff [25].

COVID 19 presented many challenges to the dental profession in Fiji. Many participants in this study experienced feelings of frustrations among team member and were concerned about the risks they were exposed to while seeing patients. This was a cause of dissatisfaction for them. According to Mishra et al. [29] concern relating to being infected with the COVID-19 contagion was one of the most frequent sources of stress among DHPs in Chhattisgarh, India and further elaborates that dentistry is such a profession that involves working in close proximity to the oral cavity and its secretions, and includes procedures that are aerosol producing is unavoidable in most of the dental procedures due to which the risk of getting infected is heightened thus leading to anxiety and frustrations. Similar findings were made by Gohil et al. [30] where fear of contagion, subjective overload, and perceived job insecurity and loss of income are causing distress among the DHPs. Some participant’s expressed that COVID has reduced their workload which allowed for some feeling of satisfaction. Similar findings were made by Prasetyo et al. [31] where Indonesian DHPs were found to have significant association with job stress reduction leading to a higher satisfaction level. However, Celik et al. [32] concluded that DHPs in the public sector examined reduced number of patients as compared to private practitioners during the pandemic. This led to DHPs being dissatisfied with the management of the pandemic. A few participants mentioned not being able to practice a full range of dental services and were only doing emergency extractions only. Ahamadi et al. [33] reported similar strategies implemented in Iran to decrease the risk of spread of contagion, the majority of non-emergency procedures we halted.

According to many studies, income is a major determinant for job satisfaction [7, 9, 29]. Income has been identified as one of the major dissatisfying aspects of this profession. Since dentistry is an expensive field to study, it is only fair to get a return on your investment made in terms of the type of pay you’d get when one starts working. In this study, majority participants felt dissatisfied as they are earning less than they invested. Some respondents reported being in debt as they are unable to repay their loans based on the salary they get. Similar findings were made by Puryer et al. [34] where 43% of DHPs were concerned with personal debt after paying for tuition fees.

Feelings of satisfaction with supervisors were reported by majority of the participants. Some of the satisfying aspects they found were their supervisors to be very helpful, understanding, good team leader, professional, supportive and engaged. Supervisor support plays a substantial role in increasing employee job satisfaction [35]. Similar finding were made by Luzzi et al. [36] that relationships with patients, colleagues and staff contribute to job satisfaction and further pointed out that there is compounding benefits to having an effective and stable dental team.^21^ Thus, in terms of the job satisfaction dimensions explored within this study, working in an effective and stable team may contribute to increased satisfaction on the relationships with colleagues, patients and staff dimensions as practices become more productive environment. Healthy relationships between superiors and their staff significantly contributes to the quality of workplace performance and has social impact which positively moderates the association between healthy workplace interactions and job stress.

Professional development is an essential aspect of an employee’s career [28]. It is critical for maintaining a stable employment and ensure growth is not stagnated [4]. For any proficient employee, the need to upskill and grow is a major priority [28]. Majority of the participants feel that there is no growth, and the profession is bottle necked. They also added that due to dentistry being an expensive field, they had to take up non – clinical course as there are not many post graduate courses available locally which limits staff from progressing in their career which led to dissatisfaction. This indicates that there is a passion among DHPs to acquire new skills and knowledge in the field of dentistry, but due to the lack of postgraduate and specialists training locally most are opting for modules and courses outside their field of work [6]. This also indicates poor design of the dental curriculum and also that the output product is short of necessary standards and competencies desired by these professionals [27]. There is a need to broaden the dental profession spectrum from being merely a graduate program [8]. Those that have upskilled, are dissatisfied as well as are limited to the types of services they can provide at their organization. This result is similar to the findings of a Romanian study where majority of the DHPSs who had post-graduate training (which is a key factor for career development) were dissatisfied [22]. Further to this Kumar et al. [1] study showed that the majority of the Pakistani DHPs who worked in the public sector were dissatisfied with the professional and career development opportunities they received during their professional life. Literature indicates human resource in health sector needs continuous training and refresher courses. Training is known to increase the self-confidence and self-esteem of employees and improves the quality of service that would significantly elevate the morale of employees in the organization [37]. Contrary to these findings, other studies suggest that those with postgraduate and specialist trainings are more satisfied than those with a degree or diploma [3, 6, 8, 19].

### Limitations

Findings of this research must be interpreted within the context of its limitations due to qualitative design; this study is limited to DHPs providing prosthetic services in Fiji, study findings included only DHPs working at MoHMS and FNU. It would be ideal for future researchers to include DHPs who offer prosthetic services in the private sector. Due to the setting of the study and the nature of the study participants, timing of data collection was limited. Data was collected via a virtual platform called Zoom based at a time convenient for the participants.

## Conclusion

To date, this has been the only study conducted exploring the perceptions of job satisfaction amongst DHPs in Fiji using qualitative analysis. The results of this study can be used to inform DHPs, their employers and academic institutes about what factors affect job satisfaction of individuals working within this profession. Factors such as nature of work, work perspectives of participants, work environment, limitations, Covid 19, income, professional relationships, and professional development were found to contribute to job satisfaction.

The empirical findings from the study indicate that DHPs employed by MoHMS and FNU, are most satisfied with their teamwork and the relationship they have with their colleagues and co-workers, followed by the nature of the work and the supervision they received. They however, indicated that they are less satisfied with professional development opportunities and least satisfied with the pay they receive and organizational support they receive. The entire structure of education and career progression in dentistry and dental technology should be addressed as a matter of urgency to achieve SDG 3, in order to encourage individuals both to join and remain within the profession.

## Electronic supplementary material

Below is the link to the electronic supplementary material.

Supplementary Material 1

Supplementary Material 2

## Data Availability

The data that supports the findings of this study are available on request from the corresponding author.

## Declarations

### Author details

^1^School of Dentistry and Oral Health, Fiji National University, Fiji.

^2^ School of Public Health and Primary Health Care, Fiji National University, Fiji.
