# Supplementary material for: Perceptions of dental health professionals (DHPs) on job satisfaction in Fiji: a qualitative study
Source: BMC Health Serv Res. 2022 Oct 18;22:1261. doi: 10.1186/s12913-022-08620-z (PMC9579612; doi:10.1186/s12913-022-08620-z)
Supplement: Supplementary file 2 — Supplementary Material 2 [file 12913_2022_8620_MOESM2_ESM.docx]

Appendix 1

**Sociodemographic Questionnaire**

| Participant no. |  | Tick box |
| --- | --- | --- |
| Gender | Male |  |
|  | Female |  |
| Race | Indo Fijian |  |
|  | ITaukei |  |
|  | Asian |  |
|  | Pacific Islander (specify) |  |
| Age | 20-30 |  |
|  | 31- 40 |  |
|  | 40- 50 |  |
| Qualification |  |  |
| Years of Experience |  |  |
| Location of practice | Ministry of Health and Medical Service |  |
|  | Fiji National University |  |
| Dental Specialty | Dentist |  |
|  | Dental Technician |  |

**Appendix 2**

**Job Satisfaction Questionnaire**

1. Think of your present work. What is it like most of the time? How well would you describe your work?
2. Think of the pay you are receiving now. How well does it justify for the work that you do?
3. Think of your supervision and organizational management you are receiving now. How would describe your present treatment?
4. Think of your opportunities for promotion that you have now. How well would you describe your present opportunities for promotion?
5. Think of the majority of the people that you are working with at the moment. How would you describe these people, your work environment and teamwork?
6. Think of your opportunities for career and professional development. Do you feel you have adequate support from your organization for development?
7. Think of some recommendations you would suggest to improve your satisfaction levels. How would you these recommendations impact your work and satisfaction levels?
